# “Fix the system … the people who are in it are not the ones that are broken” A qualitative study exploring UK academic researchers’ views on support at work

**DOI:** 10.1016/j.heliyon.2023.e20454

**Published:** 2023-09-29

**Authors:** Helen Nicholls, Danielle Lamb, Sonia Johnson, Paul Higgs, Vanessa Pinfold, Jo Billings

**Affiliations:** a Division of Psychiatry, University College London, London, United Kingdom; b Department of Applied Health Research, University College London, London, United Kingdom; c The McPin Foundation, London, United Kingdom

## Abstract

Recent evidence suggests that it remains difficult for academic researchers to preserve global well-being when working in the UK higher education sector. Our study aimed to explore academic researchers' perspectives on how they feel their mental health and well-being could be better supported within the UK higher education system. Using a combination of semi-structured and narrative interviewing techniques, we gathered the perspectives of 26 researchers. Narrative and reflexive thematic analysis were then used on the data collected. Our findings highlight the need to tackle systemic issues such job insecurity and unrealistically high workloads, given the risk they can pose to researchers' mental health and well-being. Our findings also highlight the key influence of managers and supervisors in creating a supportive environment, and the importance of going beyond *what* support is offered. That is, it is vital to effectively promote any existing or emerging support systems, and to be proactive in offering this support. Given the diversity identified in researchers’ individual situations, it is important that support is flexible and takes into consideration individual requirements and preferences. Higher education authorities and institutions need to determine how they can foster a healthy, caring environment for researchers working in this sector going forwards.

## 1 Introduction

The welfare of researchers working in higher education (HE) remains a concern. Reports continue to emphasise a taxing environment characterised by job insecurity, a “publish or perish” culture, unmanageable workloads, and an intense competition for research income that can negatively impact working relationships [1,2]. In the 2021–2022 academic year, 233,930 academic staff were registered as working across HE institutions in the United Kingdom (UK); of whom 33% were reported to be on contracts that are fixed term [3].

Expectations to participate in “Performance-based Research Funding Systems” (i.e., the UK's Research Excellence Framework (REF)) can exacerbate these pressures, and restrict academic freedom [4,5]. The REF consists of a “peer-review process” which examines the “value” and “impact” of research generated by UK HE institutions [4]. The results of the REF then help to determine how government funds are dispersed amongst these institutions [4]. Having to modify their research output in order to meet the criteria set out by the REF has left some researchers feeling discontented, as they felt less able to pursue their own research ideas and interests [5].

Ultimately, these work environments can leave researchers stressed, struggling with work/life balance, and at risk of experiencing psychological distress [6]. Citing work-related mental health difficulties, 51% of 7600 post-doctoral researchers had considered a different career [7]. Hazell et al. [8], found that doctoral researchers (or “PhD students”) in the UK reported greater levels of anxiety and depression compared to other workers.

As the evidence base continues to evolve, there has been some activity and movement towards boosting academic researchers’ mental health and well-being at work. Individual-level interventions, such as “well-being services”, counselling, mindfulness classes, and yoga classes, have been piloted in Western universities [[9], [10], [11]]. Despite showing some promise in improving well-being [12], the long-term efficacy and acceptability of these interventions is rarely evaluated, and their ability to address the root causes of stress in the HE environment has been called into question [10]. Indeed, a UK survey of 2046 “academic and academic-related staff” (including those with research responsibilities) revealed that structural interventions designed to tackle the origins of workplace stress were perceived as more helpful than individual-level interventions [11].

Various HE authorities in the UK are taking steps towards systemic, structural change. Universities UK [13] has set out a plan to integrate mental health support systems into HE institutions for staff and student populations. The “Future Research Assessment Programme”, now launched in the UK, endeavours to find new ways to make research evaluations less onerous and conducive to a healthier research culture [14]. This may include rewarding honesty and collaboration in research, as well as impact and value [15]. Nevertheless, the degree to which current structural initiatives have led to improvements in the mental health and well-being of researchers at a grassroots level, is difficult to determine (thus far). Recent evidence suggests that it remains challenging to preserve well-being in the context of the HE system [16], particularly in light of the COVID-19 pandemic [10]. The HE system cannot change overnight, but it continues to present a risk to researchers' mental health and well-being, suggesting a need to further explore what constitutes more rapid and effective support from a researchers’ point of view.

Using a “multi-method qualitative approach” [17], we aimed to explore academic researchers' perspectives on how they feel their mental health and well-being could be better supported within the UK HE system. We also explored researchers' HE journeys in-depth, to provide added context and to account for varied personal and professional characteristics. The UK HE system is the focal point of this study, however, the results may be of value to individuals associated with HE globally, given some similarities in academic culture to other countries [9], and given the UK's strong global contributions to science and research [18].

## 2 Materials and methods

### 2.1 Participants and recruitment

This study was registered with the University College London Research Ethics Committee (Ref. 21043/001). Advertisements for the study were placed on the social media platform Twitter, and in a London-based university newsletter. A snowball sampling technique was also used wherein the study was promoted through the research teams' and study participants’ own networks. Individuals were encouraged to contact the research team should they wish to take part. Individuals were eligible to participate if: (1) they currently worked (salaried or funded) at a UK university or UK university affiliated research institute; and (2) they carried out research as part of their job role. Doctoral researchers (otherwise known as PhD students) are an important part of the research workforce and were therefore also eligible to participate in the study. We sought the experiences and views of final year doctoral researchers (rather than those in the earlier stages of completing their PhD) in particular, as we considered that they would be in a better position to reflect on the entire doctoral journey. We excluded individuals who did not work at a UK university or UK university affiliated research institute, and individuals who were without research responsibilities. Doctoral researchers not in their final year of study were also excluded.

To gather a diverse range of experiences and views, we purposively sought to recruit academics from across multiple disciplines, career stages and UK universities. We also sought demographic diversity in terms of ethnicity, gender, and age. We were “pragmatic” in our approach to sample size, and we continually examined the quality of the data throughout the data collection period - aiming for a sample that was adequate in both “breadth” and “depth” [19].

### 2.2 Data collection

A full participant information sheet and link to an online consent form were shared via email with individuals who indicated an interest in taking part in the study. All participants gave informed consent before partaking in an interview. The questions in the interview schedule were developed by considering both the existing literature on this topic area [6,20], and other literature examining support experiences [21]. The questions were refined and finalised through multiple research team discussions, and following the first few interviews.

The interview consisted of a narrative interview and a semi-structured interview (please see supplementary material for the full interview schedule). The purpose of the narrative interview was to enable participants to lead the discussion on what was important to them when thinking about their mental health, well-being, and working in HE. A question asking how participants define the terms well-being and mental health was also included in this stage, given that definitions can vary contextually and individually [22]. The semi-structured interview was designed to capture researchers' views on what helps or hinders feeling effectively supported at work, maintaining well-being at work, their experiences of existing support (if applicable), and their suggestions, hopes, and expectations for support going forwards.

All interviews were conducted remotely via Microsoft Teams or Zoom and were digitally audio-recorded. The interviews were conducted and carefully transcribed by the first author - any potentially identifiable information was removed in the transcription process.

Sociodemographic data was collected via questions attached to the online consent form and through questions asked at the beginning of the interview. Data gathered included participants' age range, gender, ethnicity, the number of years they had been working in academia [23], current job title, academic discipline, whether or not they are affiliated with a Russell Group university, the geographic location of their employing university, and whether or not they belonged to a university-affiliated research institute or centre. To gather information on gender identity and sex, we used guidelines provided by the Office for National Statistics [24]. This information was gathered to add further context to participants’ experiences and was stored separately from the anonymised interview transcripts.

### 2.3 Data analysis

Two qualitative approaches were used to help interpret and understand the dataset. First, a reflexive thematic analysis was conducted to search for key patterns across the dataset. The interview transcripts were repeatedly read by the first author to achieve familiarity with the data [25]. Using NVivo Pro 12, the first author then coded all interview transcripts independently, before collapsing these codes into initial themes. The initial themes were then reviewed and amended by the research team. The development of the codes and themes occurred through examining material contained within the interview transcripts only, making the analysis inductive and exploratory.

Second, a narrative analysis was undertaken on two purposively selected interviews. The selection of the two interviews was handled by the first author with help from members of the wider research team. The two interviews selected were chosen to help better our understanding of academic job role characteristics, work relationships and university-based support, and how these factors are positioned and comprehended in singular narratives that concern mental health and well-being in HE [26]. For each of the two transcripts, the key narrative elements of the participant's story were noted by the first author. This included identifying the “core narrative”, “tone” and “narrative genre” in both stories [[27], [28], [29]]. These notes were then collated into narrative “summaries” [17], which were then distributed to the wider research team, and to the two participants, for further comment.

### 2.4 Quality and reflexivity

In line with the standards expected of qualitative research, members of the research team met at different time-points throughout the study to discuss its design and initial findings. This helped to identify any assumptions or oversights held by individual team members. To further check the “credibility” [30] of our results, the initial findings resulting from the reflexive thematic analysis were presented to research colleagues at an academic conference and to peers in a research lab meeting. The results of the narrative analysis were sent to the two participants for further thoughts/comment.

We purposefully attempted to recruit participants with varied sociodemographic and work-related characteristics, to help improve the “transferability” [30] of our results.

Finally, reflexivity is intrinsic to qualitative research. Here, we report the characteristics of the research team who conducted the study. HN is a doctoral researcher with qualitative research experience and an interest in staff well-being in HE settings. DL is a senior research fellow, SJ and PH are professors in the discipline of psychiatry, JB is a consultant clinical psychologist and associate clinical professor, and VP is a research director and co-founder of a mental health research charity. All have substantial experience in qualitative and mental health research. Although there are differences in work setting, career stage and gender, it is worth noting that the research team were all from a White ethnic background and working within the discipline of social or medical sciences.

## 3 Results

Twenty-six academic researchers took part in the study. Interviews were conducted between September 2021–February 2022 and lasted between 43 min to just over 2 h. The majority of participants reported being from a White ethnic background. The location of participants’ employing institutions ranged from London, East England, the Midlands, North West England, to Scotland and Wales. Further participant sociodemographic characteristics are presented in table one.

### 3.1 Reflexive thematic analysis

Six themes were identified using reflexive thematic analysis. The six themes and the sub-themes contained within them are reported in Table 2. They are then expanded upon below with extracts from participant’ transcripts. The subthemes are italicised within the text. Participant’ quotes have been amended to include correct grammar. Repeat words and ‘filler’ words such as ‘um’ or ‘uh’ have also been removed. Participant accounts were rich and diverse, containing a variety of experiences and a wealth of ideas. Here, we have primarily focused on the themes most pertinent to understanding and supporting their mental health and well-being in the HE environment.

**Table 1:** Participant characteristics.

Gender	
Female	20
Male	6
Age	
18 - 24	1
25 - 34	15
35 - 44	4
45 - 54	4
55 - 64	2
Number of years spent working in academia	
1–2 years	2
3–5 years	8
6–10 years	10
11–20 years	5
21–30 years	1
Role	
Research assistant	1
Final-year doctoral researcher	6
Postdoctoral researcher	1
Research associate	4
Research fellow	5
Senior research fellow	2
Senior technician	1
Lecturer	1
Senior Lecturer	3
Assistant professor	1
Associate professor	1
Discipline*	
Psychology	11
Biosciences	3
Sociology, Social policy & Anthropology	7
Education & Teaching	1
Health & Social Care	2
Medical Sciences	1
Allied Health	1
Currently affiliated with a Russell Group university?	
Yes	15
No	11
Currently working within a university-affiliated research institute?	
Yes	14
No	12

*Participants' disciplines were categorised according to the Common Aggregation Hierarchy (CAH) framework. More information can be found on the Higher Education Statistics Agency website; https://www.hesa.ac.uk/support/documentation/hecos/cah (see Table 1).

**Table 2:** Inductive themes and subthemes identified.

Themes	Sub-themes
***1. “We're not machines”*****A flawed system**	1.1 A lack of stability 1.2 Continually reaching for excellence: *“You've got to always be pushing yourself forwards academically”* 1.3 Inequality, discrimination, and isolation 1.4 The consequences: disillusionment and a negative impact on mental health and well-being
***2. “We wouldn't all be here doing it if we didn't enjoy it in some way or didn't get some sort of reward out of it”*****Factors that aid survival**	2.1 Autonomy 2.2 Why academia? Passion, feeling a sense of achievement, and being able to help others 2.3 A sense of security and not being overloaded 2.4 Individual coping strategies 2.5 Social support 2.6 Effective support provided by a higher education organisation
**3. Work relationships and the importance of*****“having somebody on the inside that you trust”***	3.1 The power of work relationships - a risk or a protective factor 3.2 Supervisors and managers *“they make or break it really”* 3.3 The perspectives of managers and supervisors
***4. “It's putting a band-aid on essentially what is a wound that needs like operating on”*****The limitations of existing support offered by higher education organisations**	4.1 Accessing existing support: concerns, uncertainty, and finding the time *4.2 “Fix the system … the people who are in it are not the ones that are broken”* “The gap” between existing support and the needs of researchers
***5. “We need to take care of our researchers”*****Expectations, hopes, and suggestions for future support**	*5.1 “Employee assistance … access to counselling … that's a realistic expectation”* Researchers' expectations for support 5.2 The importance of encouraging and fostering positive work relationships 5.3 A need for greater practical support 5.4 Beyond *what* support is offered: the importance of being proactive, and effectively promoting and monitoring the support offered *5.5 “You just have to find what works, I think, for you”* One size doesn't fit all
**6. The impact of COVID-19**	–

#### 3.1.1 “We're not machines” A flawed system

Although it was noted that work experiences can vary depending on the institution or lab a researcher presides in, (and that some of these issues are not necessarily unique to HE), many of the participants in this study highlighted that academia can be a difficult environment to navigate. This theme reflects some of the key issues encountered, the first being, *a lack of stability*. The earlier stages of a research career in particular appear to be characterised by unmet “basic needs” such as having a stable place to live/work and a steady source of income that meets the cost of living. Insecure job contracts, expectations from funders to have moved between academic research institutions (“… they all seem to want you to have moved around as if you can't progress your research from just staying in one place” [senior technician]), and often inadequate PhD stipends, were primarily cited for these needs going unmet. Greater stability or a permanent job was, understandably, a desirable objective. However, career paths both inside and outside of academia were often described as being shrouded in mystery. This sense of mystery was compounded in particular, by a lack of openness and uniformity when it comes to academic employment policies and promotions. Ultimately, as highlighted by a final-year doctoral researcher, academia was seen as “… not an easy sort of place to remain unless you-, you are lucky”, surrounded by the right people and opportunities.

Often, when thinking about achieving greater stability or progression in academia, researchers noted an “expectation” to be *continually reaching for excellence: “You've got to always be pushing yourself forwards academically”*. A senior research fellow highlighted the weight many researchers felt to singly cope with, and excel at, the multitudinous tasks expected of them within research, teaching, and/or student support roles:

“… to publish, to bring in funding, to do all of those things on top of all the extracurricular things that we're supposed to do for our CVs, I think there's just an inordinate amount of pressure and expectation on people … you could just do your job to the minimum, but then you wouldn’t progress in your career”

This culture of excellence and expectation, when coupled with job precarity in particular, was associated with a competitive work climate and an “atmosphere of … overwork”. Incidents of self-criticism or doubt also arose if researchers perceived themselves to be falling short of what was expected. Some researchers described a hesitation and/or an inability to take breaks from work as a result of this culture: “So, taking two weeks of leave, doesn't feel like you could just relax because you're just thinking of what is not being done” [research associate].

Nevertheless, the effort researchers made to reach for excellence across a variety of roles was not always recognised or rewarded. Indeed, participants noted that “success” in HE was strongly linked to what is “countable” [31], such as publications or grants awarded. Many participants spoke of a misalignment between the deadlines, expectations, and workloads set by HE authorities, compared to the amount of time and energy they were able to give:

“I think the people higher up in the university, they don’t appreciate how much time and effort goes in to doing things well and so then they overload you, so we've got a workload model which is woefully inadequate” [assistant professor]

Descriptions of *inequality, discrimination, and isolation*, also featured across researcher’s narratives, with some participants voicing that the HE system appears geared towards those who are already “advantaged”: “… the reality is the only academics who can manage to be in the job are people who have come from-, they've got money already” [research associate].

Some female participants and participants from a working-class background described experiencing incidents of misogyny and discrimination: “… he had made some very sexist comments” [assistant professor]). Some also described a sense of isolation, often due to the absence of clear representation in HE at different grades. This led to a greater risk of overwork and imposter syndrome:

“… I don't feel good enough … it’s awful. And … I'm not saying that people from a middle-class background don't-, that they feel good enough, but I think it's harder if you are from a class background that's not represented in university” [senior lecturer]

Indeed, some female researchers felt that academia “still leans towards the male perspective” [research fellow]. Reasons for this “leaky pipeline” [32] were thought to center primarily on academic culture being unconducive to taking maternity leave and childcare responsibilities. As an associate professor recounted:

“… funders who then proclaim to at times, you know, we’re wanting to take a stand and only fund institutions that have a silver in Athena Swan. But your deadlines mean that people are going to work through their holidays and that’s predominantly going to affect women … they do the bulk of the childcare”.

Some participants also wondered if efforts to improve diversity in HE could be broadened:

“… I'm not saying that there shouldn't be a focus on gender but … the people that have benefited from this focus on gender is actually middle-class women … whereas other people have got left behind quite a bit” [senior lecturer].

Further expanding on the finding of inequality, researchers working in a teaching-focused university did not always feel acknowledged for their work. Also, some researchers without a clinical professional background working in a clinical discipline, and “early career researchers” described feeling at a disadvantage when it came to applying for funding:

“… early career researchers are so disadvantaged. So, what I'm finding now is that I'm coming up with all the ideas … but I can't be the P.I. or the lead researcher … How do you develop if you're having to give ownership of your ideas to somebody else?” [research associate]

Being on the boundary between staff and student could cause feelings of isolation among doctoral researchers, as could the often “personal” nature of completing a PhD: “… it goes back to that fact that doing a PhD is very personal and therefore very isolating because you haven't got that many people to share specific experiences with” [final-year doctoral researcher].

Many researchers spoke of the *consequences* of encountering a combination of the issues above, which included *disillusionment and a negative impact on mental health and well-being*. A final-year doctoral researcher sums up a level of disenchantment experienced by some researchers: “… it has made me to an extent disillusioned in academia … all these disadvantages that people deal with”. Several researchers also described experiencing stress/burnout, sleep difficulties, depression, anxiety, and an exacerbation of existing health issues. Although, some explicitly perceived these difficulties to be sub-clinical: “… I suffer from stress and being anxious in a non-clinical way like the next person , and I think there are elements of academia that really bring that out” [research associate], whilst others noted the difficulty in pinpointing the cause of these difficulties, particularly if challenges outside of work are experienced at the same time. Researchers also described little opportunity for self-care, less time to engage with family, friends, and hobbies, and delays in achieving other life goals such as buying a house or starting a family.

#### 3.1.2 “We wouldn't all be here doing it if we didn't enjoy it in some way or didn't get some sort of reward out of it” factors that aid survival

This theme highlights key factors that helped researchers to cope with the more negative aspects of the system in which they work, a prominent factor being *autonomy,* flexibility, and a level of freedom over work schedules, duties, and research topics. Nevertheless, a senior technician reflects on the dilemma of developing one's own research interest's versus taking what job opportunities are available for stability: “… a massive negative impact on it [mental health], would be having to move into a field for which I have very little interest whatsoever”.

Despite some researchers initially going down a different career path or being unsure of the career path they wanted to take, when asked *‘why academia?’*, many spoke of a *passion* for their job role, *feeling a sense of achievement, and*/or *being able to help others*. Researchers often highlighted the fulfilment that can come from progressing their discipline, and positively impacting individuals or the wider population through their research, teaching, or student support role (even if these roles can be emotionally demanding at times: “Violence research … the job is-, it can become emotionally heavy” [research fellow]). For some, conducting these roles could also positively re-direct their focus away from personal challenges unrelated to their work. However, a research associate depicts concerns that the academic system can exploit their passion:

“Academics often do work very long hours and do so because they're passionate about their work … that shouldn't be taken advantage of, and it completely is inside academia”

A smaller number of researchers described having a *sense of security and not being overloaded* in terms of their workload, which helped to aid their survival: “Whatever happens with my research, I should still have my teaching salary … that's given me a level of security that I honestly never thought I'd have in academia” [lecturer]

When it came to further coping with some of the pressures of working in academia***,*** *individual coping strategies* and social support were often the first strategies employed or reached for. Individual strategies included exercise, persevering through uncertain or challenging moments at work, and not having an email app downloaded onto their phone. Whilst social support from friends and family could aid in taking a break from work, a senior lecturer also said: “… I notice my family just always say ‘just leave it for a bit’, and it's like, well, they don't understand. I can't leave it for a bit”.

Counselling provided through universities was the primary form of *effective* support *provided by a higher education organisation* mentioned by researchers, as it helped with understanding and coping with the symptoms of anxiety, stress, and depression, whether these symptoms arose from personal or work-related challenges.

Careers advice and support directed towards those in the earlier stages of their career, was also described as particularly beneficial. Examples included presentations which helped to demystify the academic career path, access to an early career researcher coaching scheme, and the presence of:

“… internal funding mechanisms … I think that is just hugely invaluable to-, especially early career researchers. It gives you a foot on the ladder to say that I'm actually-, I'm getting some practice writing proposals and getting some money” [research fellow]

Also found to be of benefit was the offering of seminars and conferences which encourage collaboration, the offering of conflict resolution services and, the offering of “well-being days”. With regards to the impact agenda, a research fellow said: “it takes the pressure off publishing your *Nature* papers … some of the work just won’t-, is not going to be suitable for those journals”.

#### 3.1.3 Work relationships and the importance of “having somebody on the inside that you trust”

*The power of work relationships* as either *a risk or a protective factor*, was depicted by the majority of the interviewees. Having trusted colleagues in the workplace, whether a fellow researcher, a safety officer, or a member of the administration team, aided with the disclosure of any mental health difficulties experienced, with problem solving and creativity, with boosting mental health and well-being, and with the sharing of good practice and resources: “… she put together an equality, diversity, and inclusion support resource pack … it's the most amazing resource I think I've ever had access to since I joined” [senior lecturer]. A research associate further highlighted how the presence of a flatter hierarchy can be particularly protective for those in the earlier stages of their career: “It’s a difficult terrain to navigate, and like friends are only knowledgeable so far, but if you've got somebody who's higher up, who's like fighting your case, that can be really supportive”. Nevertheless, the competitive climate created by the HE system was noted as one of the primary factors preventing these collaborative and positive work relationships: “… the amount of time people trying to scoop one another, it's just-, it's just toxic” [final-year doctoral researcher].

Directly related to work relationships as a risk or a protective factor, was the impact of *supervisors and managers* in either helping or hindering a researchers' mental health and well-being. Given the more direct sway they could have on lab or department culture, workloads, autonomy, providing support and training around research processes, and achieving career goals, it appeared that: “*they make or break it really”.* A research fellow highlights the positive impact of their manager’s flexibility:“… I was spending the Friday doing my thesis … but then she gave me another day a week to do that … So that sort of flexibility from a manager's point of view made it a lot less stressful than it could have been”, whilst a senior research fellow highlights the negative impact of a very difficult work relationship:

“My supervisor was very difficult to work with … she pretty clearly blamed me for not trying hard enough and she would shout a lot … I was pretty confident that the study, just, you couldn’t recruit to it, but I was worried about her negative perception of me and how that would impact me in the long term … in one meeting, I quit my job and my PhD”

For some researchers, managers'/supervisors’ high workloads were thought to be a contributing factor to less effective management, whilst others believed this could be down to a lack of management training.

A senior research fellow sums up *the perspectives of managers and supervisors*, who often described the role as “rewarding”, and who also mentioned being aware of (and trying to mitigate) the challenges faced by those they manage:

*“* I make sure that I'm incredibly flexible in terms of whatever she needs to do with, you know, family and home life. Because … she's a young woman who's coming into research, and I know what it's like … I have a lot of responsibilities at home and I know how difficult that can be”.

#### 3.1.4 “It's putting a band-aid on essentially what is a wound that needs like operating on” the limitations of existing support offered by higher education organisations

With regards to *accessing existing* support, researchers described *concerns, uncertainty, and finding the time*. As depicted by a research associate, concerns primarily centred on the information flows of disclosures, particularly, whether a disclosure about a colleague may get back to them: “… we're her only students. So, if he goes to her and says students have been complaining, it's essentially the same thing as like us going to her and complaining”. A research fellow also highlights concerns that the disclosure of a mental health or well-being-related difficulty may impede career progression: “… they might be in the first place saying yeah we are very supporting … but … if they were to choose between you and another person, they might choose another person. I may be wrong, but it's better not to risk it”.

Uncertainty surrounding what university-offered support currently exists, and who it is for, was common. This uncertainty was exacerbated by a lack of effective signposting (e.g., email advertisements that blend “into the wallpaper”), and not having the time to pursue accessing the support offered, due to high workloads. For others, the uncertainty stemmed from not having felt that they needed to look for, or access, university-offered support for their mental health or well-being. The perceived severity of the mental health or well-being difficulties they experienced also played a role in whether or not researchers felt the support available would be either open to them, or effective:

“I don't feel like I have a right to access things like the university counselling because … you’re not someone who doesn't need any support, but you’re not, kind of, so bad that you need a lot of support” [final-year doctoral researcher]

*“Fix the system … the people who are in it are not the ones that are broken”* sums up “*the gap” between existing support and the needs of researchers*. Many researchers perceived some of the individual interventions offered such as mindfulness, art, or mental health workshops, as “tokenistic” and limited in their ability to tackle systemic issues or difficult work relationships which can lead to poorer mental health and well-being. This gap was also reflected in some of the more negative experience’s researchers had had, with regards to university-offered support. For some researchers, inexperienced facilitators that existed in university counselling spaces and occupational health services meant that this support was described as not as effective as it could have been. In some cases, as highlighted by a lecturer below, researchers also felt unable to re-disclose mental health difficulties or other work-related challenges when there was a lack of action from the university in following through with suggested or promised support: “… it was a very, very near miss … to tell them once that I needed that help was one thing, but I didn't feel the following week, you know, that I could do that again”.

Interestingly, some researchers from a psychology discipline also wondered if mental health departments could do more to promote a mental health friendly culture in the department: “… given that is the work that we do” [research associate].

#### 3.1.5 “We need to take care of our researchers” expectations, hopes, and suggestions for future support

The following quote - “Employee assistance … access to counselling … that's a realistic expectation” **-** encompasses many researchers' expectations for support. Suggestions were made as to how these services could be made more effective such as, having shorter waiting times, arranging the availability of more sessions, being matched with the right counsellor/facilitator, implementing cognitive behavioural therapy approaches, and ensuring that the service in general has an idea of the types of difficulties researcher's face. For some researchers, thinking about expectations and hopes for support led them to reflect on the discrepancy between staff and student support:

“I think that they should offer at least some kind of, not necessarily counselling service, but someone you can talk to confidentially if you need to. But again, they do this for students, but I haven't seen anything like that for staff” [senior technician]

Ultimately, it was seen as important to have someone trained and “accessible” to talk to about any mental health/well-being difficulties encountered.

Incidentally, whilst questions were raised as to whether a sense of community and good work relationships can be “artificially” created, *the importance of encouraging and fostering positive work relationships* could not be overstated by some researchers when it came to positively influencing mental health and well-being at work. Enabling relationships to bloom between those in the earlier stages of their career and more senior researchers (for example, through considering office layouts) appeared to be particularly important, as senior researchers were looked to as key influencers with regards to encouraging collaboration, rejecting expectations to overwork and normalising having a life outside of academia.

Suggestions related to improving management and supervision included having “… dedicated roles within an academic department for staff well-being or management” [lecturer] or “… having a sort of separate manager, a place to go, which is just there for development” [senior lecturer]. Providing training for managers and supervisors on how to effectively conduct these roles was suggested, however, questions were raised as to the extent to which mental health training for managers could be imposed from the top-down:

“… people resent training because it just adds to their workload … if you send a bunch of scientists on a kind of course that they perceive as being a bit fluffy, it will just-, they will just resent it” [lecturer]

Another key comment related to fostering positive connections at work included promoting and encouraging diversity in the workplace:

“… visibility is important. We need to have female professors, we need to have ethnic minority professors, we need to have openly gay professors, things like that so that you can see that academia is for everyone … it is getting better, but it’s still got a way to go.” [assistant professor].

A further quote by the assistant professor: “… actual genuine investment in people” summed up *a need for greater practical support* inclusive of (but not limited to); an end to insecure job contracts, a stipend/salary/pension reflective of expertise and time dedicated, greater recognition for the amount of work taken on and individual strengths, and better advice and support for pursuing careers inside and outside of academia - so that researchers can see a path for progression. As suggested by a lecturer: “… have a career structure where it's OK to be a postdoc for 20 years, if that's what you want to do”, or “… allow some flexibility where they could do a week's work shadowing of somebody in industry or something different”.

The assistant professor goes on to highlight researchers' views on the need to foster stronger relationships and levels of communication between senior leaders and themselves, the need to tackle inadequate workload models/unrealistically high workloads (in this case, by hiring more people), and the need for a greater level of openness when it comes to promotions:

“I think that kind of feeling like the people higher up are actually listening because it doesn’t feel like they are at all, and if you say we’ve got poor well-being amongst our staff, they’ll just put an online course on, that you’ve then got to try and find 2 hours to do, which you don’t have anywhere because you’re overworked. So I think … more bodies on the ground, you know, I think that would make people feel a lot more supported. And more transparency as well because a lot of the times in academia it feels like people get promoted or held back for very obscure reasons, you know, and its bizarre”

Practical suggestions related to doctoral researchers primarily centred on treating them more as members of staff (for example, clearly stating their annual/sick leave entitlement). With regards to changes that could be made by funders, a research associate said: “… this rule from funder's that you can't be a PI if you don't have a permanent job, because it's just a cycle of never being able to develop”, whilst a senior lecturer mentioned including a section on the application form dedicated to how researcher mental health and well-being will be managed during a study: “I think there should be a section where … you outline almost like your exit plan if something-, if you needed time off”. There were also calls for the UK government to provide greater investment in research and education in general. Nevertheless, as highlighted by an associate professor, it was at times difficult to determine if practical, systemic change was possible, and who might be responsible for setting these changes in motion: “… you almost have to break the system down again and rebuild it differently, but I don't know if anybody knows how you would even do that”. The need for more practical support also extended to role/research topic specific scenarios. This included having “the structures and the training and the supervision” in place to enable researchers who work in emotionally challenging research areas (such as violence or mental health research) to develop necessary “clinical skills”.

Many researchers went *beyond* what support *is offered*, and discussed *the importance of being proactive, and effectively promoting and monitoring the* support *offered*:

“… they can have fantastic policies, you know, everyone's got policies. But unless you have a committee that monitors how the policy is implemented, you may as well not have it” [final-year doctoral researcher]

Good organisational support was thought to be proactive and offered more than once: “I honestly think we should be having more check ins” [senior lecturer]. The importance of effectively promoting the support offered was also discussed. Specifically, that confidentiality should be highlighted and advertisements need to be memorable. When promoting support, some researchers also felt there was a need to clarify what is meant by key support terms such as mental health, well-being, or reasonable adjustments. The latter is particularly important, given the subtle differences in the ways in which researchers defined mental health and well-being:

“… I think mental health to me is more of a clinical thing … a mental illness or problem …” [research associate]

“… mental health is just literally how-, how you're coping …” [research assistant]

On a similar vein, some researchers felt that there was a need for universities to actively help alter the “clinical perception” attached to mental health and/or well-being support – through explicitly highlighting that the support offered is for everyone.

*“You just have to find what works, I think, for you” One size doesn’t fit all* highlights the importance of taking into consideration individual needs and preferences. Some researchers felt that effective support could involve universities and other HE authorities giving researchers the time, space, and means to do things which are good for their own personal mental health and well-being:

“… You might be better off just saying to people, we’ll-, we'll pay for you to have a day out walking in the Peak District or … like you know-, something that is not-, it doesn't feel like an administration task, it actually feels like we care about you looking after yourself” [research fellow]

Indeed, the research fellow goes on to sum up below that what works for some in terms of work patterns and supporting their mental health and well-being, will not always work for others:

“… there'll be people that are quite like vociferous about don't do emails outside of work time … don't set these expectations … And then you have other people replying saying, well, I do those things and it's a bit like … you just have to find what works, I think, for you. And that will … depend partly on your family situation and your priorities and that sort of stuff.”

#### 3.1.6 The impact of COVID-19

The COVID-19 pandemic was described as having exacerbated the lack of stability (as a result of delayed promotions, reduced funding opportunities, delays in the completion of existing studies), and appeared to further highlight the inflexibility of academia when it comes to reaching for excellence: “I was trying to be like a human who was living through an unprecedented global event and it didn't feel like there was room for that in-, in academia” [final-year doctoral researcher].

Work from home (WFH) rules meant it was often harder to transition between work and home life, particularly for those with caring responsibilities: “… it might be like go down for a little break, transition to daddy mode, and then ten minutes later … I've got to go back to work … which is still in the house” [research fellow]. There was also a decrease in formal and informal work conversations that can boost mental health, well-being, creativity, and problem solving. However, for others, WFH rules somewhat improved supervisory relationships: “… it formalized our meetings with her … we would start to see her on a more regular basis on Zoom” [Research associate] and enabled better engagement in some work-based or personal activities of value: “… I was just sort of clearing things that I've been meaning to write for a long time” [postdoctoral researcher].

The pandemic also highlighted a general lack of practical support for researchers, with some mentioning difficulties with securing funding extensions and a lack of help when it came to setting up their WFH space. The general negative ramifications of the COVID-19 pandemic on personal mental health and well-being were also noted outside of the context of work. Some researchers understandably noted heightened feelings of isolation, anxiety, and concern as they watched the pandemic unfold across the world.

### 3.2 Narrative analysis

Following being sent their respective narrative summaries, the two participants reported that they were happy with what had been written, and that these summaries captured their stories thus far. Pseudonyms have been used. The two narrative summaries are presented below.

#### 3.2.1 Case study 1 – survival

For Ricky, a researcher in the earlier stages of his career, at the core of his narrative was the importance of good “people skills”. Ricky gave multiple examples throughout his narrative of where ineffective and effective communication hindered and helped his mental health, respectively. Perhaps the most significant form of ineffective communication described was within the managerial/supervisory relationship. Ricky described a “series of events” which: “… just kind of built up and built up and built up because neither of us had the correct communication skills to deal with one another”. To improve their relationship, both Ricky and his supervisor engaged with informal support provided through the university.

Ricky perceived the support offered as effective: “It worked really well”. He described the positive transformation they had both gone through with regards to their communication skills, with the university-offered support appearing to act as a catalyst for this growth: “It has taught me a lot … a lot of people skills that I don't think I would have been able to learn anywhere else”.

Differing communication styles found within university-offered counselling spaces also featured within Ricky’s narrative. When first accessing counselling, he encountered a space which allowed him to better understand his current feelings and past experiences. However, upon accessing the support for a second time, he described the experience as “probably the worst kind of turning point” for his mental health. The facilitator appeared to be at the heart of why this experience was negative, as they were perceived to be not actively engaging and communicating with him:

“… the session became just about suicidal thoughts … this series of questions which just felt completely out of place from what I said before and also out of place from the answers I was giving”

When thinking about how researchers could be better supported, Ricky understandably stressed the importance of training managers and supervisors, especially with regards to developing their communication skills around mental health:

“The whole management training, and wellness and mental health training in understanding not only what the university can provide, but how to approach certain situations … how to cope with others …”

For Ricky, the narrative genre of his account seemed to be that of a ‘survivor’. Ricky detailed the “roller coaster” he experienced with regards to his mental health as he navigated personal challenges, his research, work relationships, and university-based support. Nevertheless, Ricky ultimately described his journey as being: “very much a success story …, you know, I’ve been able to solve all this stuff , especially with the counselling”.

The central tone of Ricky's narrative was ‘reflective’, due to his comprehensive interview answers, his clear ordered version of events, and his thoughtful manner during the interview.

#### 3.2.2 Case study 2 – connected and disconnected

For Maxine, a senior researcher, her core narrative centred on the importance of “connectedness”. Whilst having a permanent job was a significant factor that contributed positively to her well-being at work, it was also her sense of connectedness within her immediate environment which enabled her to feel valued, able to progress, and feel well in herself. At the centre of this feeling of connectedness was her strong relationships with her colleagues and her manager, all of whom were noted to be aware of, and respectful of, her responsibilities outside of work. A passion for her job role within the department she worked for, also contributed to her sense of connectedness:

“… there is definitely that feeling of connectedness to my team and also to the [department] … it helps with my well-being to really believe in what I’m doing and to share the values, I think, that are generally shared by the people that are working on … research in the way we are … people understanding that I have a home life and respecting that …”

Her experiences of connectedness within her immediate environment starkly juxtaposed with the disconnection she had felt and observed within the academic system as a whole. The system was ultimately depicted as one which is not accommodating of researcher’ needs: “… academia in general have created a system whereby people are often overworked, at times can feel undervalued and very stressed and on precarious contracts … it's a broken system”.

Her experiences of connection and disconnection were not only reflected in what was said during the interview, but also through the tone of her narrative. When describing her immediate work environment, the narrative tone was that of passion and warmth. However, the tone of her narrative switched to frustration -“bullshit”, “fucked-up system” - when describing the academic system as a whole.

The narrative genre encapsulating Maxine's story was ‘a call to action’. Whilst she highlighted that existing psychological services and strong relationships between colleagues play a role in supporting researchers at work, she was clear that, ultimately, systemic change needs to happen which bridges “the gap” between the needs of researchers and the ways in which various 10.13039/100001174HE authorities (for example; policy makers, funding bodies and senior management) operate:

“… But to be honest with you, what really needs to happen is that the system needs to be changed because it's a bit like we can provide people with … all these solutions for mental health problems, let's stop giving them frigging mental health problems in the first place, like let's pay people adequately, let's give them permanent contracts and make them feel valued and make them feel secure …”

## 4 Discussion

Using qualitative approaches, we explored academic researchers’ perspectives on how they feel their mental health and well-being could be better supported within the UK HE system. The systemic issues highlighted by the researchers in our study were consistent with those evident in existing literature [1,2,6]. Many of the researchers in this study indicated that systemic (and often practical) change, needs to accompany those factors which aid their survival in the academic environment, such as autonomy. Suggestions for systemic change included an end to insecure job contracts, embedding equality, diversity, and inclusion (EDI) training and policies in work culture that effect real change in this regard, more realistic workloads, and clear, accessible routes for career progression. Case study two in particular reflects the strong, emotive views some researchers had around implementing tangible, systemic change to better support their mental health and well-being at work. However, as highlighted by those who took part in our study, it can be difficult to determine who might be responsible for directing these changes. Some participants in our study, and existing research, have found that it is a “shared responsibility” [research fellow], with recommendations directed towards different individuals, HE authorities and stakeholders [11,33,34]. Nevertheless, the views of particular HE stakeholders and authorities, inclusive of senior management in universities, funding bodies, and academic policy makers, have not been well-explored. It is imperative to qualitatively explore their views on systemic change and mental health/well-being in HE (in addition to the views of researchers, academics, and other HE groups), to identify any discrepancies, and to ensure that mutual objectives can be worked towards. Fostering stronger relationships and levels of communication between researchers and these other elements of the HE system is particularly important, as, for those who took part in our study, it was felt that the challenges they encountered were not always heeded or acted upon. It is critical to communicate where positive systemic change is being strived for (an example being the “Future Research Assessment Programme”, UKRI, 2022), given the recent exacerbation of systemic issues as a consequence of the COVID-19 pandemic, and the increasing number of researchers who are considering leaving HE. A recent survey of 7000 UCU members found that 75% of individuals with research responsibilities were “likely” to depart HE [35].

The key influence of immediate managers and supervisors has been highlighted by this study and others [36,37]. Training managers and supervisors on how to manage effectively was essential for some of the researchers in the present study. Despite this, Wellcome [2] found that only 48% of managers in research institutions had received management training. Concerns over mandating management training were understandably raised in this study, particularly as researchers often described being over 100% capacity in terms of their workloads. That said, case study one does depict how university-offered support which teaches effective communication can have a positive impact on these types of relationships, and, in this case, subsequently improve the researchers’ mental health.

Related to participants’ suggestions of having more than one manager, is the concept of mentoring. Mentoring is not a novel suggestion, however, proffers and occurrences of mentorship are not ubiquitous [38]. Mentorship can help to foster relationships between early career researchers and senior researchers, and can aid in keeping “underrepresented” groups in the science workforce [39]. Being able to access the support of a trusted senior researcher could promote tendencies to discuss mental health/well-being difficulties or promote disclosures of mistreatment such as bullying. The latter could be particularly important, given the “power” dynamics that can exist between early career researchers and their immediate management or principal investigator [40].

Being proactive with offering mental health or well-being support is vital, given that this study and others [41] have identified uncertainty surrounding university-offered support (often due to poor signposting, and poorly defined key support terms such as ‘mental health’ ‘well-being’ and ‘reasonable adjustments’).

There was also diversity in researchers’ individual situations. Similar to Jackman et al. [18], this is perhaps best demonstrated in the context of COVID-19. Where some researchers mentioned a poorer work life balance as a result of WFH rules, others commented on an ability to partake in important hobbies and pursuits they would not normally have been able to do. As such, there still needs to be a level of flexibility when it comes to support, ensuring that individual requirements and preferences are accounted for. To further echo Jackman et al. [18], immediate and senior management in universities, and other HE authorities, need to determine how they can foster a healthy, caring environment for researchers working in the HE sector going forwards. The below recommendations created from this research are not exhaustive, however, we hope they can be considered in conjunction with the recommendations listed across other existing relevant literature, to help better support researchers at work.

• There needs to be stronger communication from senior management, funders, national governments, and other HE authorities about how systemic issues such as job precarity, challenges related to EDI, and unrealistically high workloads are being tackled, and any progress made. If this work is not being carried out, those with the power to effect change must consider how to do so.
• Increase the visibility and accessibility of existing support. University-offered support needs to be explicitly confidential; it needs to be promoted in a memorable way; and key support terms such as ‘reasonable adjustments’ and ‘mental health/well-being’ should also be clearly defined.
• Further development of management and supervision skills. This could be achieved through providing relevant training and supervision for supervisors/managers. Providing researchers with the option of having more than one manager, or a mentor, could also be considered.
• Encourage an open and compassionate working culture, so that those who want to, feel comfortable sharing their own experiences of mental health or well-being difficulties. Particularly senior members of staff, who can help to positively influence working culture.
• Provide more opportunities for careers advice and support. Help de-mystify career paths both inside and outside of academia.
• Implement check ins and follow ups with researchers to help identify any emerging support needs proactively, whether these relate to careers, mental health, or well-being. Check in's and follow ups could be carried out by managers/supervisors, occupational health services, or mentors.
• One approach does not fit all. Support offered needs to be flexible, balancing organisational/institutional needs and goals, with the needs and preferences of individuals.

### 4.1 Strengths and limitations

This study had both strengths and limitations. Steps were taken to boost the “credibility” and “transferability” [30] of our findings, including discussing and amending initial findings as a team. Using two qualitative approaches also allowed us to search for patterns across the dataset, whilst also exploring in more depth how the themes slotted into personal stories. Nevertheless, we acknowledge that the experiences and views contained within the two cases selected for the narrative analysis may not be representative of the researchers interviewed in this study, nor researchers within UK HE as a whole [26].

Whilst the study sample was diverse in a number of ways including in terms of career stage and UK university type, the sample did not differ greatly in terms of ethnic background, gender, and discipline – potentially limiting how transferable our results are. Reasons for the lack of diversity in these areas and thus limited transferability of our findings, could be partly due to our use of a snowball sampling technique.

Researchers from ethnic minority backgrounds and those working in a humanities discipline, for example, may experience different challenges and have additional or different support needs that are not captured in this study.

## 5 Conclusions

The challenges facing researchers who work in HE are multi-faceted and complex, as are their support needs. Relationships between researchers and other HE authorities and stakeholders need to be strengthened, to better communicate what challenges are being encountered, and where positive change is being strived for (or implemented). Immediate management plays a key role in how a researcher experiences their working environment, and it is important that managers and supervisors are given the tools and the time to look after their own well-being, as well as the well-being of those they manage. Whilst experiences and therefore support needs can vary individually, it is important that any support offered is proactive, and that it is advertised in a memorable and clear manner.

## Author contribution statement

Helen Nicholls: Conceived and designed the experiments; Performed the experiments; Analyzed and interpreted the data; Wrote the paper.

Danielle Lamb, Sonia Johnson, Paul Higgs, Jo Billings: Conceived and designed the experiments; Analyzed and interpreted the data; Commented on drafts of the paper.

Vanessa Pinfold: Conceived and designed the experiments; Analyzed and interpreted the data.

## Data availability statement

The authors do not have permission to share data.

Supplementary content related to this article has been published online at [URL].

## Funding statement

This publication was made possible through funding provided by a Bloomsbury Colleges Studentship in partnership with The McPin Foundation (Grant reference: ES/P000592/1). This report is independent research supported by the National Institute for Health Research ARC North Thames. The views expressed in this publication are those of the author(s) and not necessarily those of the National Institute for Health Research or the Department of Health and Social Care.

## Declaration of competing interest

The authors declare that they have no known competing financial interests or personal relationships that could have appeared to influence the work reported in this paper.
