# Supplementary material for: “Fix the system … the people who are in it are not the ones that are broken” A qualitative study exploring UK academic researchers’ views on support at work
Source: Heliyon. 2023 Sep 29;9(10):e20454. doi: 10.1016/j.heliyon.2023.e20454 (PMC10582291; doi:10.1016/j.heliyon.2023.e20454)
Supplement: Multimedia component 1 [file mmc1.docx]

Interview Topic Guide

**Initial form(s) to complete:**

During the first stage of the interview, an initial icebreaker/warm-up conversation will be held. During this stage, the interviewer will complete a form with the participant which gathers data around participants’ current general job title, academic discipline, university type & location, and research institute status. If participants have not had a chance to fill out the sociodemographic information form (which is attached to the consent form) prior to the interview, this will be completed with the participant here also.

**Narrative interview:**

*During this next stage of the interview, we hope to hear about your experiences as an academic researcher. If you feel comfortable to do so …*

- Would you be able to describe to me any events throughout your career as an academic researcher so far which significantly relate to your personal mental health or well-being, in either a positive or negative way?

*Prompts:*

- *What led you to begin an academic research career?*
- *Do you think you will remain in an academic research career?*
- *Are there any events or experiences that you remember which particularly shaped, or affected your identity as an academic researcher? (Question removed after the 3^rd^ interview due to confusion over how to respond to it)*
- *What comes to mind when you think of the term(s) well-being or mental health?*
- *If you feel comfortable to do so, would you be able to talk me through any mental health difficulties you may have experienced during your career as an academic researcher so far?*
- *How would you say these difficulties have impacted on, or been impacted by, your work as an academic researcher?*

**Semi structured interview:**

*This next stage of the interview will involve me asking more specific questions in order to explore your expectations for mental health support, your thoughts on maintaining positive well-being at work, and your views on what is needed in order to effectively support academic researchers’ mental health and well-being.*

**Maintaining positive well-being at work:**

- Is there an aspect of your job that you find particularly effects (or helps or hinders) your ability to maintain positive well-being at work?
- Are there some things that you have personally found helpful in terms of maintaining positive wellbeing at work?

*Prompts:*

- *At a disciplinary level*
- *In terms of the wider job context*

**Barriers/facilitators to feeling supported:**

- Are there any factors that you think can either help or hinder an academic researcher from feeling supported at work in terms of their mental health or well-being?

*Prompts:*

- *At an individual level*
- *In terms of work relationships*
- *In terms of the wider job context*

**Expectations/hopes for support:**

- Is there any sort of mental health support you would expect to be offered by an academic institution?
- Is there any sort of mental health support you would hope to be offered by an academic institution?
- Have you previously experienced support for your mental health or well-being whilst working at an academic institution?

If yes:

**Support offered** (Gee et al., 2022)**:**

- Could I ask a little bit more about [support] and what [it] entailed?
- Were you offered any other types/level of support? - Did they offer to contact any services outside of the university?
- Were you happy with the support that was offered to you?
- “Is there anything you wish you were offered with regards to support?”
- Did it meet your expectations?

**Changes within the higher education system:**

- Are there any aspects of the higher education system that you feel currently work well in terms of supporting academic researcher’s mental health or well-being?
- What do you think is needed in order to effectively support academic researchers’ mental health and well-being going forwards?

*Prompts:*

- *At a government level*
- *At an institutional level*
- *At an individual level*
- *Funder’s responsibilities*
- *The higher education systems’ overall engagement with other significant parties such as the public or media.*

**Closing:**

- Is there anything else you would like to add before we finish?
